# Predictors of the Acceptance of an Electronic Coach Targeting Self-management of Patients With Type 2 Diabetes: Web-Based Survey

**DOI:** 10.2196/34737

**Published:** 2022-08-16

**Authors:** Zeena Harakeh, Hilde Van Keulen, Koen Hogenelst, Wilma Otten, Iris M De Hoogh, Pepijn Van Empelen

**Affiliations:** 1 Department of Child Health TNO, Netherlands Organization for Applied Scientific Research Leiden Netherlands; 2 Department of Training and Performance Innovations, TNO Netherlands Organization for Applied Scientific Research Soesterberg Netherlands; 3 Department of Microbiology and Systems Biology, TNO Netherlands Organization for Applied Scientific Research Zeist Netherlands

**Keywords:** mobile health, type 2 diabetes, self-management, eCoach, Technology Acceptance Model

## Abstract

**Background:**

Type 2 diabetes (T2D) is a lifestyle-related disease whose prevalence increases with age. Diabetes self-management through mobile health (mHealth) apps enables patients with T2D to improve their health. According to the Technology Acceptance Model (TAM), technology acceptance (ie, intended use) is necessary to ensure mHealth can be implemented successfully. Therefore, the specific acceptance requirements of patients with T2D should be considered.

**Objective:**

This cross-sectional study aims to examine the extent to which different TAM predictors are associated with the acceptance of a diabetes app including an electronic coach (eCoach; Iris app) among patients with T2D.

**Methods:**

Using a web-based survey, data on 92 patients with T2D (mean age 62.76 years, SD 8.29 years) were collected. Acceptance of the Iris app with the TAM predictors (ie, perceived usefulness, perceived ease of use, social influence, perceived self-efficacy, perceived security, prior usage experience, perceived health, and propensity of data/information sharing) was assessed. Further, control variables (ie, gender, age, education, ethnicity, household, BMI, amount of years with diabetes, diabetes-related complaints, and medication use) were assessed.

**Results:**

Multiple linear regression analyses showed that acceptance of the Iris app was positively associated with perceived usefulness (β=.57, *P*<.001), social influence (subjective norm; β=.20, *P*=.004), and willingness to share data (β=.25, *P*<.001). In addition, acceptance regarding the Iris app was higher among patients with T2D with overweight (β=.23, *P*=.01) or obese BMI (β=.21, *P*=.01). The model explained 75.8% of the variance in the acceptance of the Iris app by patients with T2D. In addition, perceived usefulness of the Iris app was positively related to perceived ease of use (β=.32, *P*<.001), subjective norm (β=.26, *P*=.004), perceived control (β=.19, *P*=.03), willingness to share data (β=.20, *P*=.01) regarding the Iris app, and perceived security regarding general use of apps/smartphone/internet (β=.15, *P*=.04). The model explained 58.2% of the variance in patients’ perceived usefulness about the Iris app.

**Conclusions:**

Among patients with T2D, the belief that the use of the Iris app is helpful/beneficial, the willingness to share their Iris app data, and others’ approval of using this app can stimulate the acceptance of this app. In addition, the belief that the use of (health) apps is reliable and secure, the belief that the use of the Iris app is easy to use, a higher perceived capability and personal control with using this app, the willingness to share their Iris app data, and others’ approval of using this app can stimulate the perceived usefulness of such an app. These TAM predictors explained a high variance in acceptance and perceived usefulness of the Iris app. Implications for practice are addressed.

## Introduction

### Background

Type 2 diabetes (T2D) is a global public health problem leading to increased mortality and morbidity risk. Furthermore, T2D can affect patients socially and economically [1]. The prevalence of this chronic disease is high and still increasing [2,3]; in particular, people older than 50 years are at an increased risk [4-6]. In addition, T2D prevalence is higher among people with low socioeconomic status (SES) [7]. Empirical evidence indicates that a healthier lifestyle (eg, eating healthy and more physical activity) and monitoring blood glucose levels may improve the health status of patients with T2D and reduce health complications of diabetes [5,8]. For societal, economic, and ethical reasons, increasing demands are made on individuals to self-manage their own health and to maintain a healthy lifestyle. Patients with T2D are supposed to take control over their life and health, and diabetes self-management is therefore crucial [9].

For patients with T2D, mobile health (mHealth) apps can be a valuable tool to support self-management [9,10]. In general, the advantages of mHealth apps include a wide reach of people, and tailored and timely health information, education, and support [11]. Regarding T2D, many apps have been developed over the past years, which focus on supporting self-management and education of patients with T2D to promote a healthy lifestyle and health [4,11,12]. However, the elements of these apps vary, and may include insulin management applications, wearable blood glucose meters, automated SMS text messages, health diaries, and virtual health coaching [11]. The meta-analysis of Greenwood et al [9] showed that apps including components of 2-way communication, personalized data, and tailored education and feedback contributed most to an improved HbA1c (also referred to as glycohemoglobin or hemoglobin A1c, an indicator of adequate diabetes management). Previous empirical studies showed that the usability and efficacy of these T2D apps vary to a great extent [13]. Technology acceptance is crucial to ensure mHealth, such as these T2D apps, can be implemented as planned, and thus understanding the requirements for this acceptance is very important. The few empirical studies that examined the predictors of patients’ acceptance of diabetes management showed that predictors based on extended versions of the Technology Acceptance Model (TAM; [14]) explained a high variance (around 60%) in patient’s intention to use diabetes management apps, which is a proxy for acceptance [15]. To anticipate the development of the Iris (T2D) app, which is intended as an electronic coach (eCoach) to support self-management of patients with T2D, the aim of this study is to determine which predictors are associated with acceptance of this specific app (for a description of the Iris app, see the “Methods” section).

### Prior Research

The TAM [14] is one of the most prevailing, dominant theoretical models that has been frequently applied to predict consumer acceptance of health technology such as mHealth [16]. The TAM is based on social-cognitive models such as the Theory of Planned Behavior [17,18], Diffusion of Innovations Theory [19], and Social Cognitive Theory [20]. According to the original TAM, behavior (in this case, using the Iris app) is determined by behavioral intention, which is a proxy for acceptance. Furthermore, the following 2 major cognitive predictors, perceived usefulness and perceived ease of use, directly predict acceptance of health technology such as the Iris app. Perceived usefulness refers to an individual’s belief that the use of this technology is helpful/beneficial, whereas perceived ease of use refers to an individual’s belief that this technology is easy to use [14,16].

In the course of the years, the original TAM has been modified by extending it with additional predictors. Several extended TAM models have been proposed. For example, the Unified Theory of Acceptance and Use of Technology (UTAUT; [21,22]) included social influence (ie, subjective norms) as another important cognitive factor that predicts acceptance of health technology. Subjective norms refer to an individual’s belief of how other people, especially the people whom they trust and resort to, will evaluate them when using the technology [21]. In addition, Cialdini [23] has emphasized the importance of social norms and made a distinction between injunctive norms (ie, an individual’s belief of what most other people approve of) and descriptive norms (ie, an individual’s belief of what most other people typically do). Thus, social influence in the TAM can refer to injunctive and descriptive social norms, where subjective norms are a person’s perception of injunctive norms of relevant other persons [24]. The UTAUT [21], Senior Technology Acceptance Model (STAM; [25]), and an extension of the TAM by Fathema et al [26] included perceived self-efficacy/behavioral control as a cognitive factor predicting perceived usefulness, perceived ease of use, and acceptance. Perceived self-efficacy/control refers to an individual’s belief to successfully handle technology and personal control over technology [26]. Other examples of extended versions of the TAM included perceived security and trust (ie, the extent to which a user believes that a particular service is secure), prior usage experience with mobile phones and eHealth, perceived health (ie, self-management of diabetes), and willingness to share data (ie, willingness to share personal information/data; eg, [16,21,25,27-40]). Although several predictors have been proposed according to (extended) TAM models, only a few empirical studies examined TAM predictors of diabetes management apps and showed support for these predictors [15]. Nevertheless, empirical studies that tested the TAM predictors regarding acceptance of T2D eCoaching apps, specifically, are lacking. Previous studies focused predominantly on the usability and efficacy of T2D apps [13] or used a qualitative approach to examine the acceptance of diabetes apps (eg, [41]).

### Study Objective

The aim of this study was to examine the predictors associated with the acceptance of the Iris app for patients with T2D. According to (extended) TAM models, we hypothesize that the following predictors are positively associated with acceptance of the Iris app: perceived usefulness, perceived ease of use, social influence (ie, descriptive and subjective social norms), perceived self-efficacy/behavioral control, perceived security, prior usage experience, perceived health, and willingness to share data. In line with others (eg, [21,24]), we included the following control variables: sociodemographic factors (ie, gender, age, education, ethnicity, household) and health-related factors (ie, BMI, amount of years with diabetes, diabetes-related complaints, and medication use).

## Methods

### Participants

Participants provided informed consent prior to completing the 20-minute survey. Inclusion criterion was men and women with T2D who had a smartphone that they used regularly. We used convenience sampling. Recruitment took place from July to November 2018 through an advertisement on the website of the Dutch Diabetes Association [42], through flyers handed out at the National Diabetes Challenge festival in Amsterdam (September 2018), and through a Facebook advertisement in November 2018. The online survey was an “open survey” that was voluntary and accessible for individuals who received, through these advertisements and flyers, the link to the study information and survey online. In total, 97 participants filled out the online survey. The individuals who provided digital informed consent filled in all items on the questionnaire. Thus, the participation rate equaled the completion rate. As a reward, participants received an online web shop voucher of €10 (US $10.43).

### Procedure

The recruitment materials included a link that referred interested individuals to the study information online (including information about the duration of the survey, incentive, data storage, the research team, and the purpose of the study) and a subsequent consent page. Following digital consent, participants could continue to fill in the survey. The 20-minute survey consisted of questions regarding their experience with (diabetes) apps and their willingness to share health information with others. Privacy and anonymity of the participants were guaranteed.

### The Anticipated App Iris

The Iris app is a dynamically tailored intervention that provides personalized diet and physical activity advice, as well as behavioral support. Users start with intake: First, users fill out health data (eg, BMI, medication usage, HbA1c). Second, users can decide whether they are willing to work on a physical activity or diet goal. To provide personalized dietary advice, users are asked to rate from different food products (eg, fruits, vegetables, sugar-sweetened beverages) their average intake per week (quantity per day and number of days per week). Similarly, when users select a physical activity goal, they are asked to rate their activities on a general week (physical activity toward work, at work, around the house, leisure time, and sports). Based on the health data, users are provided with a recommended diet (Mediterranean, low carbohydrate, or low caloric diet). Based on the dietary intake or physical activity data, users are provided with feedback on what food categories or physical activities are compliant with the recommended intake, and which categories could be improved. Next, users can self-set a specific daily or weekly goal (eg, eating 2 pieces of fruit for 3 days).

When a goal is set, a user will be asked to daily assess whether they have reached their goal, and assess their goal motivation, goal competence, and mood (each on a 3-point scale: negative, neutral, or positive). The app includes a monitoring page, where people can monitor the number of times they reached their goal for 7 days. In addition, based on whether a goal was (partly) reached or not, each week a user is provided with (positive) feedback on their performance. Furthermore, based on the daily diary data, the most important barriers for not reaching a goal are assessed (motivation, competence, planning, or mood), and a tailored intervention is recommended to overcome the barrier, based on an effective behavior change technique.

### Measurements

**Figure 1:**
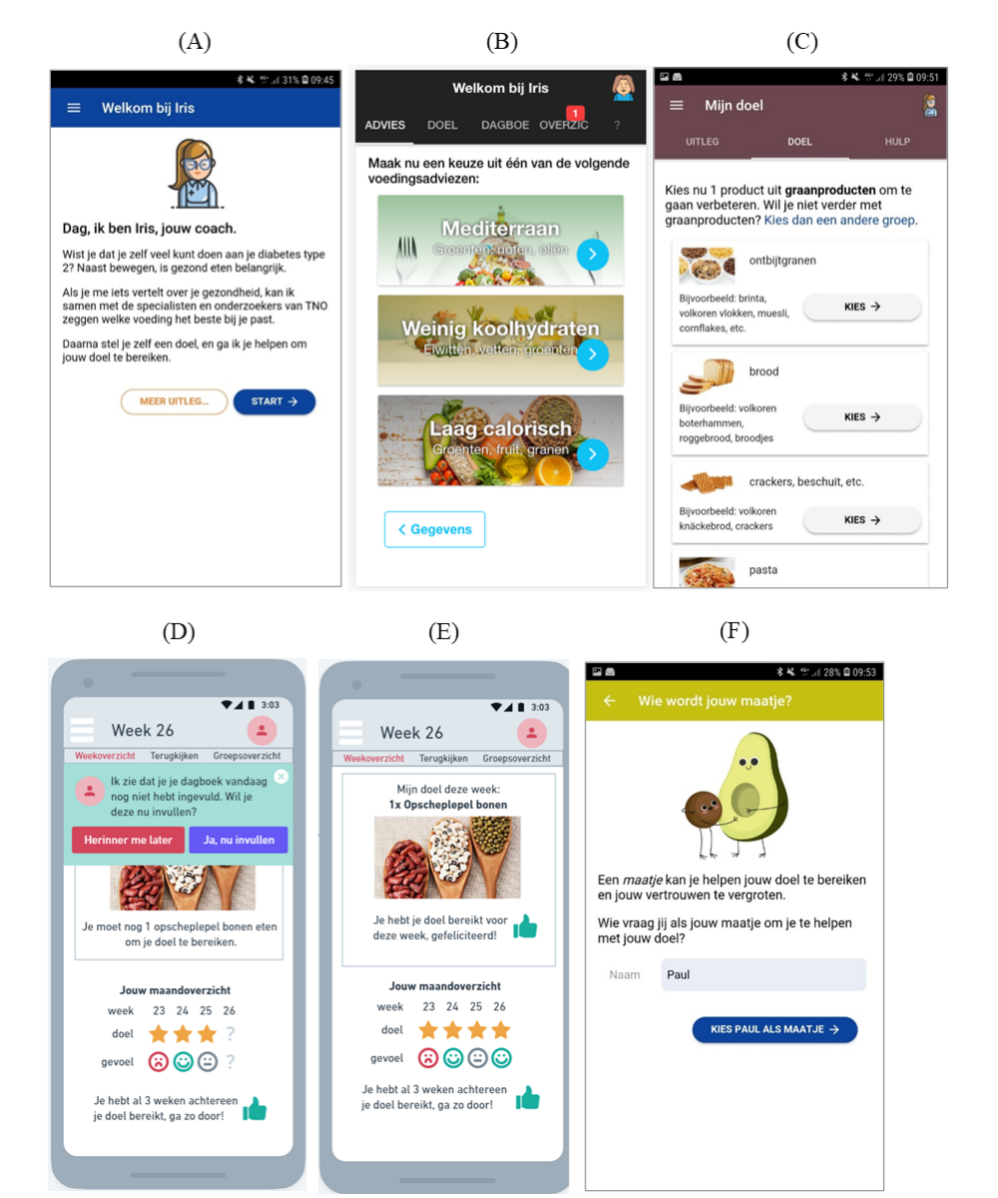
Screenshots of the Iris T2D app. (A) Introduction of the coach Iris. (B) Nutritional advice. (C) Setting daily dietary goals. (D) Daily reminder. (E) Feedback and compliments. (F) Tips and exercises.

The majority of the measurements were self-constructed and based on theoretical reasoning (original and modified versions of the TAM), empirical studies (eg, [16]), and the results of the 3 focus group interviews we conducted regarding the barriers and facilitators to use the Iris app (n=23 patients with diabetes). The outcome variable was technology acceptance. Predictors of technology acceptance included perceived usefulness, perceived ease of use, social influence, perceived self-efficacy, perceived security, prior usage experience, propensity of data/information sharing, and perceived health. According to the TAM, these predictors can align with the use of mHealth technology in general (eg, apps, smartphone, and internet) as well as to a specific mHealth technology (eg, a specific app such as the Iris app). In our study, we distinguished these TAM predictors. To examine the specific predictors of the Iris app, we provided participants with mock-ups of this app, explaining different elements of the potential coach, to provide participants with an idea about what the Iris app (ie, digital coach) would offer. A display with different screens of the Iris app was shown to the participants (Figure 1). We will first describe in detail below the assessment of the outcome variable; followed by the TAM predictors regarding the general use of apps, smartphone, and internet and the TAM predictors regarding the use of the Iris app; and finally the control variables. Unless otherwise specified, all questionnaire items were answered on a 5-point Likert scale ranging from 1 (strongly disagree) to 5 (strongly agree). The description of the items of the outcome variable and predictors (including Cronbach α) is depicted in Table 1. If the variable was measured with 2 or more items, the mean was used to compute the variable.

**Table 1:** Descriptive statistics of the outcome variable and TAM predictors.

Construct					Item		Cronbach α		Mean (SD)		n
**Outcome variable**											
	Acceptance of the Iris app				I would like to use the Iris app, if this was presented to me. I intend to use the Iris app to improve my health. I would use the Iris app for managing type 2 diabetes.		.936		3.67 (0.91)		N/A^a^
**Predictors regarding the general use of apps/smartphone/internet**											
	Perceived usefulness/outcome expectancies				Health apps can help me improve my health. Health apps make it easier for me to cope with my diabetes. Health apps ensure that I am less dependent on others. By daily keeping track of my diet, I am better at coping with my diabetes. I appreciate it when I receive direct advice from an app to improve my lifestyle. An app needs to be fun, if I want to use it. I like to use my mobile phone. I find it annoying to receive daily reminders of an app {R}^b^. I question whether an health app can support me quite effectively {R}.		.787		3.64 (0.69)		91
	Perceived ease of use				I find it easy to use health apps. I quickly learn how to operate new apps.		*r*=0.702, *P*<.001^c^		3.65 (1.06)		89
	**Social influence**										
		Subjective norm^d^			My doctor thinks that health apps could help me with my diabetes.		N/A		3.30 (0.95)		61
		Descriptive norm^d^			Most of the people I know already use health apps.		N/A		2.81 (1.20)		62
	Perceived security				I think it is important that information of health apps is reliable. It is important that the data I enter in the app are secure. I am confident that my data in health apps are secure. I only want to use an app if I know that my privacy is guaranteed.		.836		4.12 (0.93)		92
	**Prior usage experience**										
		Behavior regarding internet use			I use the internet to search for information about my diabetes (problems).		N/A		3.86 (1.27)		87
		Frequency of smartphone use^e^			How often do you use your smartphone?		N/A		N/A		92
		Use of app for their diabetes^e^			Do you use apps for your diabetes and which one(s)?		N/A		N/A		92
	**Perceived health**										
		Attitude regarding diabetes self-management			I am worried about my diabetes. I have my diabetes well in hand. I think that my treatment (medication/insulin) helps my diabetes. With eating healthy food, I can decrease the risk of diabetes problems. Exercising helps to reduce diabetes problems. My weight is of influence on my diabetes. My health is important for me. I would like to improve my overall health.		.730		3.65 (0.67)		92
		Self-efficacy regarding diabetes self-management			I succeed in controlling my diabetes. I succeed in eating healthy daily. I succeed to sufficiently exercising weekly. I succeed to watch out for the number of meals and snacks. I succeed in checking my blood glucose regularly.		.822		4.19 (0.57)		92
		Self-care regarding diabetes self-management			I take my diet into account for a good blood glucose level. I take my diabetes medication as required. I have tried to lose weight because of my diabetes. Once in a while I eat too much sweetness and other food rich in carbohydrates {R}. I prefer not to go to doctor appointments for my diabetes {R}. Sometimes I eat more than I intended to {R}. I tend to skip exercise/sport {R}. I am bad taking care of myself with respect to diabetes {R}.		.730		3.54 (0.71)		92
	**Predictors regarding the use of the Iris app**										
		Perceived usefulness			I like a digital coach that motivates me to eat healthier. I find it useful if a digital coach motivates me to eat healthier.		*r*=0.863, *P*<.001^c^		3.53 (0.88)		92
		Perceived ease of use			I question whether a digital coach can support me quite effectively {R}. I find it unpleasant if I would receive daily messages of a digital coach {R}. I prefer to be supported by a real person {R}. It is easier for me to cope with my diabetes with a digital coach.		.698		3.03 (0.71)		92
		**Perceived social influence**									
			Subjective norm	My health practitioner would find it important that I use the digital coach. People close to me (family, friends) would find it important that I use the digital coach.		*r*=0.568, *P*<.001^c^		3.14 (0.73)		92	
		Perceived control		I succeed in using a digital coach daily.		N/A		3.25 (1.04)		92	
		Willingness to share data		My general practitioner would be allowed to view the data that I maintain in the app. My data that I gather in the app can be used for research. I would share my data if these help other patients with diabetes.		.835		3.86 (0.80)		92	

^a^N/A: not applicable.

^b^{R} means reverse coded.

^c^Cronbach α could not be calculated for variables with 2 items, and therefore Pearson correlation (*r*) and *P* value were reported.

^d^These 2 predictors had a great number of missing information because participants responded that “they did not know” (30/92, 33% and 29/92, 32%, respectively).

^e^These 2 predictors were dichotomous; for more information about the percentage of these predictors, see Table 2.

### Overview

#### Outcome

The outcome variable was acceptance of the Iris app. Acceptance refers to the intended use of the Iris app. It was measured with 3 items. An additional response option to the 5-point Likert scale included “not applicable,” which was recoded as missing.

#### TAM Predictors Regarding the General Use of Apps, Smartphone, and Internet

Perceived usefulness was assessed by outcome expectancies that indicate the positive consequences of using health apps. It was measured with 9 items.

Perceived ease of use was assessed with 2 items.

Perceived social influence was assessed by the following 2 variables: subjective norm (also referred to as injunctive norm) regarding app use, and descriptive norm regarding app use. Subjective norm regarding app use was measured with 1 item on a 5-point Likert scale ranging from 1 (strongly agree) to 5 (strongly disagree). Answers were reverse coded. Descriptive norm regarding app use was measured with 1 item on a 5-point Likert scale ranging from 1 (strongly agree) to 5 (strongly disagree). Answers were reverse recoded.

Perceived security was assessed with 4 items.

Prior usage experience was assessed by the following 3 variables. Behavior regarding internet use was measured with 1 item. Frequency of smartphone use was measured with 1 item and response categories were recoded into 2 categories: “more than 1 time a day” and “daily to weekly.” Use of apps for their diabetes was measured with different responses (eg, Koolhydraatkenner; Mijn Eetmeter; MyFitnessPal), and computed into 2 response categories: “do not use any apps for diabetes management” and “yes, do use, 1 or more apps for diabetes management.”

Perceived health was assessed by the following 3 variables, obtained from the Diabetes Management Self-Efficacy Scale (DMSES; [43]) and the Diabetes Self-Management Questionnaire (DSMQ; [44]). *Self-efficacy regarding diabetes self-management* was measured with 5 items. *Attitudes regarding diabetes self-management* were measured with 8 items. For each item, participants could also answer “not applicable,” which was recoded as missing. *Self-care regarding diabetes self-management* was measured with 8 items. A high score represents positive self-care.

#### TAM Predictors Regarding the Use of the Iris App

Perceived usefulness was assessed with 2 items.

Perceived ease of use was assessed with 4 items.

Perceived social influence was assessed by the subjective norm regarding digital coach use with 2 items. Descriptive norm was not measured because the digital coach was not yet publicly available or in use.

Perceived control was measured with 1 item.

Willingness to share data was measured with 3 items.

#### Control Variables

##### Sociodemographic Factors

Gender was coded as “male” and “female.” Age was categorized into 2 based on the sample median: “<63 (=25-62) years” and “≥63 (63-84) years.” Education was assessed using 10 categories (ranging from primary education to university degree), and recoded into 3 categories, of which low-level education was computed as the reference category and intermediate- and high-level education as the 2 dummies. Ethnicity was based on country of birth. This variable was recoded into 2 categories: “Dutch” and “other.” Household was recoded into 2 categories: “living alone” and “living together with partner and/or children.”

##### Health-Related Factors

BMI was measured by length (in centimeters but recoded into meters) and weight (kg). We calculated BMI (kg/m^2^), and classified it into 3 categories: “normal” (≥20 and <25 kg/m^2^), “overweight” (≥25 and <30 kg/m^2^), and “obese” (≥30 kg/m^2^) [45]. We computed 2 dummies with “normal” BMI as the reference category.

Amount of years with diabetes was measured by asking how long (number of years) people were diagnosed with T2D. Diabetes-related complaints that participants could indicate included, for example, eye problems, nerve damage (neuropathy), kidney problems. Ticking more than 1 box was possible. This was computed into the following 2 categories: “no, having no complaints” and “yes, having 1 or more complaints.” Medication use was measured by asking which medicines do you use for your T2D, and was computed into the following 2 categories: “not using medication” and “using medication.”

### Data Analyses

We first checked for multicollinearity (*r*≥0.80) between the predictors, but this was not the case. The full correlation table, including correlations between the predictors, can be requested from the first author. To examine the research question, we performed the analyses in 3 steps. First, we performed bivariate linear regression analyses with the (control) variables and the outcome (step 1). Second, we conducted a multiple linear regression analysis (step 2), including the significant (control) variables of step 1. We first analyzed, in step 2a, a multiple linear regression model including the significant TAM predictors regarding the general use of apps, smartphone, and internet as well as the use of the Iris app (eg, perceived usefulness, perceived ease of use, descriptive and subjective norms, perceived self-efficacy/behavioral control, perceived security, prior usage experience, perceived health, and willingness to share data). Subsequently, in step 2b, we added the significant control variables (eg, gender, age, education, ethnicity, household, BMI, amount of years with diabetes, diabetes-related complaints, and medication use) to the multiple linear regression model. Finally, we repeated the multiple linear regression analysis with only the significant (control) variables of step 2 (step 3). We used pairwise missing data and a *P* value <.05 was considered significant. Moreover, we computed the *R*^2^ for steps 2 and 3. To provide more additional information about the model quality, we plotted the dependent variable against the predicted values, and performed bootstrapping. Overall, the scatterplot shows that the model gives a pretty good prediction for the outcome (see Multimedia Appendix 1). In addition, the bootstrapping analyses indicated that overall our model is quite robust.

### Ethics Approval

The Institutional Review Board (IRB) of the Netherlands Organization for Applied Scientific Research (ie, TNO) approved the study (IRB registration number: 2018-029).

## Results

### Demographics and Descriptive Analysis

Of the 97 participants that filled out the survey, 2 participants never used a smartphone and 1 participant had missing values on the majority of the questions, and thus, were removed from the data set and analyses. In addition, 2 other participants were excluded from the analyses, as they indicated that they did not experience any diabetes-related complaints, did not use medication, and had 0 years of diabetes, ergo they may not have had T2D. Thus, in total, 92 participants were included in the data analyses. The sample characteristics of the 92 participants are presented in Table 2. The majority of the participants (57/92, 62%) did not use an app for diabetes management. The participants that did use health or lifestyle apps predominantly used the app “MyFitnessPal” (9/92, 10%), and 2 Dutch food-related apps, namely, “Koolhydraatkenner” (10/92, 11%) and “Mijn Eetmeter” (14/92, 15%).

Descriptive analyses (mean and SD) of the predictors and outcome are depicted in Table 1. Except for descriptive norms regarding app use (mean 2.81, SD 1.20), average ratings were higher than 3 on all variables, implying a positive stand on these variables.

**Table 2:** Demographics of the study participants (n=92).

Variable			Values
**Female gender, n (%)**			48 (52)
**Age (years)**			
	Range	25-80	
	Mean (SD)	62.76 (8.29)	
	≥63^a^, n (%)	49 (53)	
	Missing, n (%)	1 (1)	
**BMI (kg/m** ^2^ **), n (%)**			
	<25 (normal)	15 (16)	
	25-<30 (overweight)	39 (42)	
	≥30 (obese)	35 (38)	
	Missing	3 (3)	
**Highest completed level of education,** **n (%)**			
	Low	40 (44)	
	Intermediate	28 (30)	
	High	24 (26)	
Country of birth (the Netherlands), n (%)			86 (93)
Household (together with partner or children), n (%)			76 (83)
Frequency of phone use (>1 time/day), n (%)			74 (80)
**Number of years with diabetes**			
	Range	0-34^b^	
	Mean (SD)	11.41 (8.32)	
	Missing, n (%)	2 (2)	
Diabetes-related complaints (yes), n (%)			80 (87)
Medication use (yes), n (%)			82 (89)
**Practitioner, n (%)**			
	General practitioner/nurse practitioner	68 (74)	
	Hospital doctor	21 (23)	
	Other	3 (3)	
Use of apps for diabetes management (yes), n (%)			35 (38)

^a^The median was used to divide age into 2 categories.

^b^There was 1 person indicating 0 years with diabetes, but also indicated medication use and complaints regarding diabetes and was included in the sample.

### Predictors Associated With the Acceptance of the Iris App

We first identified with bivariate linear regression analyses the factors that were significantly (ie, *P*<.05) associated with the acceptance of the Iris app (Table 3, step 1). The findings indicated that beliefs of patients with T2D that general and specific use of technology is helpful/beneficial (β=.42, *P*<.001 and β=.81, *P*<.001, respectively) and easy to use (β=.32, *P*=.003 and β=.55, *P*<.001, respectively) increased the acceptance of the app. Besides, their belief that using (health) apps is reliable and secure was related to an increase in the acceptance of the app (β=.29, *P*=.005). Patients with T2D who used the internet to search for information about their diabetes (β=.29, *P*=.007) or had a positive attitude toward diabetes self-management (β=.34, *P*=.001) showed a higher acceptance of the app. Except for behavior regarding internet use, prior usage (frequency of smartphone use, β=.09, *P*=.40; use of apps for their diabetes, β=.02, *P*=.84) was not significantly associated with acceptance of the app. Furthermore, perceived health (self-efficacy regarding diabetes self-management, β=–.05, *P*=.62; and self-care regarding diabetes self-management, β=–.15, *P*=.15), except for attitude regarding diabetes self-management, was not significantly associated with acceptance of the app. Beliefs of patients with T2D that others (health practitioner, family, friends) approved of using the app (β=.61, *P*<.001) and perceiving a higher capability and personal control over using the app (β=.58, *P*<.001) increased the acceptance of the Iris app. Descriptive norms regarding the general use of apps, smartphone, and internet were not significantly associated with acceptance of the app (β=.13, *P*=.32). Patients with T2D who were willing to share their app data showed an increase in the acceptance of the app (β=.52, *P*<.001). Furthermore, being overweight (β=.40, *P*=.008) or experiencing more diabetes-related complaints (β=.32, *P*=.002) was significantly associated with a higher acceptance of the app. The sociodemographic factors (gender, age, education level, ethnicity, household) were not associated with a higher acceptance of the app.

Only the (control) variables that showed to be significant (*P*<.05) in the bivariate analyses were entered in the multiple linear regression analysis (Table 3, step 2b). The findings indicated that beliefs of patients with T2D that the use of app is helpful/beneficial (β=.52, *P*<.001), that others approved of using this app (β=.18, *P*=.02), and whether they were willing to share their app data (β=.22, *P*=.002) increased the acceptance of the Iris app. Furthermore, being overweight (β=.22, *P*=.01) or obese (β=.20, *P*=.02) was associated with an increase in the acceptance of the Iris app compared with having a “normal” weight. Moreover, the multiple regression analysis that we repeated with only the significant (control) variables (ie, for those where *P*<.05) of step 2 (Table 4, step 3) showed a similar pattern of results.

As perceived usefulness of the app showed to have a strong effect, we performed additional analyses to examine the predictors related to perceived usefulness of the app. We used the same statistical procedure as we did for acceptance of the app. The findings of the bivariate analyses for perceived usefulness of the app (Table 5, step 1) showed to be similar as the findings of the bivariate analyses for acceptance of the app, except for BMI, which was not significantly related to perceived usefulness of the app (β=.24, *P*=.11). The significant (control) variables (ie, *P*<.05) were entered in the multiple regression analysis (Table 5, Step 2b). The findings were partly in line with the findings of the acceptance of the app. Similar to the findings of the acceptance of the app, beliefs of patients with T2D that others (ie, health practitioner, family, friends) approved of using the app (β=.26, *P*=.006) and their willingness to share their app data (β=.22, *P*=.01) were related to an increase in perceived usefulness of the app. In contrast to the findings of the acceptance of the app, the findings also showed that beliefs of patients with T2D that the use of (health) apps is reliable and secure (β=.24, *P*=.04), that the use of the digital coach is easy to use (β=.32, *P*=.003), and that higher perceived capability and personal control with using the app (β=.21, *P*=.03) were related to an increase in the perceived usefulness of the app. Moreover, the multiple regression analysis, which we repeated with only the significant (control) variables (ie, *P*<.05) of step 2 (Table 6, Step 3), showed a similar pattern of results.

**Table 3:** Predictors associated with the acceptance of the Iris app by means of a linear regression analyses (method=Enter).

Variable							Acceptance of the Iris app^a^																						
							Step 1									Step 2a^b^									Step 2b^c^				
							B (SE)				β (*P* value)				B (SE)					β (*P* value)				B (SE)					β (*P* value)
**Control variables**																													
	Gender (reference: male)					0.07 (0.19)				.04 (.74)				—^d^									N/A^e^						
	Age (reference: <63 years)					–0.07 (0.19)				–.04 (.71)				—									N/A						
	**Education (reference: low level education)**																												
		Middle/intermediate level of education			0.23 (0.22)				.12 (.31)				—									N/A							
		High level of education			–0.19 (0.24)				–.09 (.42)				—									N/A							
	Ethnicity (reference: Dutch)					–0.54 (0.38)				–.15 (.16)				—									N/A						
	Household (reference: living alone)					0.43 (0.25)				.18 (.09)				—									N/A						
	**BMI (reference: normal)**																												
		Overweight			0.72 (0.27)				.40 (.008)				—									0.40 (0.15)					.22 (.01)		
		Obese			0.35 (0.27)				.19 (.20)				—									0.36 (0.15)					.20 (.02)		
	Amount of years with diabetes					–0.01 (0.01)				–.06 (.60)				—									N/A						
	Diabetes-related complaints (reference: having no complaints)					0.87 (0.27)				.32 (.002)				—									0.27 (0.17)					.10 (.11)	
	Medication use (reference: not using medication)					–0.37 (0.31)				–.13 (.23)				—									N/A						
**Predictors regarding the general use of apps/smartphone/internet**																													
	Perceived usefulness/outcome expectancies					0.55 (0.13)				.42 (<.001)				0.08 (0.14)					.06 (.57)				0.09 (0.13)					.07 (.49)	
	Perceived ease of use					0.27 (0.09)				.32 (.003)				–0.07 (0.07)					–.08 (.36)				–0.11 (0.07)					–.13 (.13)	
	**Social influence**																												
		Subjective norm			0.19 (0.12)				.20 (.13)				—									N/A							
		Descriptive norm			0.10 (0.10)				.13 (.32)				—									N/A							
	Perceived security				0.29 (0.10)				.29 (.005)				–0.03 (0.09)					–.04 (.70)				–0.002 (0.09)					–.002 (.98)		
	**Prior usage experience**																												
		Behavior regarding internet use			0.21 (0.08)				.29 (.007)				0.07 (0.06)					.09 (.28)				0.06 (0.06)					.09 (.30)		
		Frequency of smartphone use			0.20 (0.24)				.09 (.40)				—									N/A							
		Use of apps for their diabetes			0.04 (0.20)				.02 (.84)				—									N/A							
	**Perceived health**																												
		Attitude regarding diabetes self-management			0.54 (0.16)				.34 (.001)				0.12 (0.11)					.08 (.28)				0.13 (0.11)					.08 (.23)		
		Self-efficacy regarding diabetes self-management			–0.07 (0.14)				–.05 (.62)				—									N/A							
		Self-care regarding diabetes self-management			–0.19 (0.13)				–.15 (.15)				—									N/A							
	**Predictors regarding the use of the Iris app**																												
		Perceived usefulness			0.85 (0.06)				.81 (<.001)				0.56 (0.10)					.54 (<.001)				0.54 (0.09)					.52 (<.001)		
		Perceived ease of use			0.70 (0.11)				.55 (<.001)				0.003 (0.11)					.003 (.98)				–0.06 (0.11)					–.05 (.57)		
		**Perceived social influence**																											
			Subjective norm	0.76 (0.10)				.61 (<.001)				0.22 (0.10)					.17 (.03)				0.22 (0.09)					.18 (.02)			
		Perceived control		0.50 (0.08)				.58 (<.001)				0.09 (0.07)					.10 (.19)				0.08 (0.06)					.10 (.20)			
		Willingness to share data		0.59 (0.10)				.52 (<.001)				0.23 (0.08)					.20 (.007)				0.25 (0.08)					.22 (.002)			

^a^Step 1 includes bivariate linear regression analyses, step 2a multiple linear regression analysis with only the significant predictors of step1, and step 2b multiple linear regression analysis adding the control variables in Block 2.

^b^*R*^2^=0.750.

^c^*R*^2^=0.782.

^d^Not available.

^e^Not applicable (ie, variable was not significant in the previous step and thus not included in the analyses).

**Table 4:** Predictors associated with the acceptance of the Iris app by means of a linear regression analyses (method=Enter).

Variable						Acceptance of the Iris app			
						Step 3^a^			
						B (SE)			β (*P* value)
**Control variables**									
	Gender (reference: male)				N/A^b^				
	Age (reference: <63 years)				N/A				
	**Education (reference: low level of education)**								
		Middle/intermediate level of education		N/A					
		High level of education		N/A					
	Ethnicity (reference: Dutch)				N/A				
	Household (reference: living alone)				N/A				
	**BMI (reference: normal)**								
		Overweight		0.41 (0.14)			.23 (.01)		
		Obese		0.39 (0.15)			.21 (.01)		
	Amount of years with diabetes				N/A				
	Diabetes related complaints (reference: having no complaints)				N/A				
	Medication use (reference: not using medication)				N/A				
**Predictors regarding the general use of apps/smartphone/internet**									
	Perceived usefulness/outcome expectancies				N/A				
	Perceived ease of use				N/A				
	**Social influence**								
		Subjective norm		N/A					
		Descriptive norm		N/A					
	Perceived security			N/A					
	**Prior usage experience**								
		Behavior regarding internet use		N/A					
		Frequency of smartphone use		N/A					
		Use of app applications for their diabetes		N/A					
	**Perceived health**								
		Attitude regarding diabetes self-management		N/A					
		Self-efficacy regarding diabetes self-management		N/A					
		Self-care regarding diabetes self-management		N/A					
	**Predictors regarding the use of the Iris app**								
		Perceived usefulness		0.60 (0.08)			.57 (<.001)		
		Perceived ease of use		N/A					
		**Perceived social influence**							
			Subjective norm	0.25 (0.08)			.20 (.004)		
		Perceived control		N/A					
		Willingness to share data		0.29 (0.07)			.25 (<.001)		

^a^*R*^2^=0.758; step 3 is multivariate analysis with only the significant predictors of step 2.

^b^Not applicable (ie, variable was not significant in the previous step and thus not included in the analyses).

**Table 5:** Predictors associated with perceived usefulness of the Iris app by means of a linear regression analyses (method=Enter).

Variable					Perceived usefulness of the Iris app												
					Step 1^a^					Step 2a^b^					Step 2b^c^		
					B (SE)		β (*P* value)		B (SE)			β (*P* value)		B (SE)			β (*P* value)
**Control variables**																	
	Gender (reference: male)				0.03 (0.18)		.02 (.87)		—^d^					N/A^e^			
	Age (reference: <63 years)				–0.14 (0.19)		–.08 (.47)		—					N/A			
	**Education (reference: low level of education)**																
		Middle/intermediate level of education		0.22 (0.22)		.12 (.31)		—					N/A				
		High level of education		–0.06 (0.23)		–.03 (.80)		—					N/A				
	Ethnicity (reference: Dutch)				–0.48 (0.37)		–.13 (.20)		—					N/A			
	Household (reference: living alone)				0.30 (0.24)		.13 (.22)		—					N/A			
	**BMI (reference: normal)**																
		Overweight		0.43 (0.26)		.24 (.11)		—					N/A				
		Obese		0.02 (0.27)		.01 (.96)		—					N/A				
	Amount of years with diabetes				0.001 (0.01)		.01 (.91)		—					N/A			
	Diabetes-related complaints (reference: having no complaints)				0.75 (0.26)		.29 (.005)		—					0.30 (0.21)			.11 (.16)
	Medication use (reference: not using medication)				–0.42 (0.29)		–.15 (.16)		—					N/A			
**Predictors regarding the general use of apps/smartphone/internet**																	
	Perceived usefulness/outcome expectancies				0.52 (0.12)		.41 (<.001)		–0.11 (0.17)			–.09 (.53)		–0.09 (0.17)			–.07 (.58)
	Perceived ease of use				0.26 (0.08)		.32 (.002)		0.003 (0.09)			.003 (.97)		–0.03 (0.09)			–.03 (.78)
	**Social influence**																
		Subjective norm		0.22 (0.12)		.24 (.06)		—					N/A				
		Descriptive norm		0.05 (0.09)		.07 (.60)		—					N/A				
	Perceived security			0.30 (0.10)		.31 (.003)		0.22 (0.11)			.23 (.05)		0.23 (0.11)			.24 (.04)	
	**Prior usage experience**																
		Behavior regarding internet use		0.18 (0.07)		.25 (.02)		–0.02 (0.08)			–.03 (.76)		–0.03 (0.08)			–.04 (.71)	
		Frequency of smartphone use		0.001 (0.23)		.000 (*>*.99)		—					N/A				
		Use of app applications for their diabetes		0.05 (0.19)		.03 (.80)		—					N/A				
	**Perceived health**																
		Attitude regarding diabetes self-management		0.38 (0.16)		.25 (.02)		–0.10 (0.14)			–.06 (.49)		–0.09 (0.14)			–.06 (.50)	
		Self-efficacy regarding diabetes self-management		–0.06 (0.14)		–.05 (.65)		—					N/A				
		Self-care regarding diabetes self-management		–0.15 (0.13)		–.12 (.25)		—					N/A				
	**Predictors regarding the use of the Iris app**																
		Perceived ease of use		0.74 (0.10)		.60 (<.001)		0.44 (0.13)			.36 (.001)		0.40 (0.13)			.32 (.003)	
		**Perceived social influence**															
			Subjective norm	0.70 (0.10)		.58 (<.001)		0.32 (0.11)			.27 (.005)		0.32 (0.11)			.26 (.006)	
		Perceived control		0.47 (0.07)		.56 (<.001)		0.16 (0.08)			.19 (.04)		0.18 (0.08)			.21 (.03)	
		Willingness to share data		0.45 (0.11)		.41 (<.001)		0.25 (0.10)			.23 (.01)		0.24 (0.10)			.22 (.01)	

^a^Step 1 is bivariate analyses, step 2a is multivariate analysis with only the significant predictors of step 1, and step 2b is multivariate analysis adding the control variables in block 2.

^b^*R*^2^=0.588.

^c^*R*^2^=0.599.

^d^Not available.

^e^Not applicable (ie, variable was not significant in the previous step and thus not included in the analyses).

**Table 6:** Predictors associated with the acceptance of the Iris app by means of a linear regression analyses.

Variable				Acceptance of the Iris app		
				Step 3^a^		
				B (SE)		β (*P* value)
**Control variables**						
	Gender (reference: male)		N/A^b^			
	Age (reference: <63 years)		N/A			
**Education (reference: low level of education)**						
	Middle/intermediate level of education		N/A			
	High level of education		N/A			
Ethnicity (reference: Dutch)				N/A		
Household (reference: living alone)				N/A		
**BMI (reference: normal)**				N/A		
	Overweight		N/A			
	Obese					
Number of years with diabetes				N/A		
Diabetes-related complaints (reference: having no complaints)				N/A		
Medication use (reference: not using medication)				N/A		
**Predictors regarding the general use of apps/smartphone/internet**						
	Perceived usefulness/outcome expectancies		N/A			
	Perceived ease of use		N/A			
	**Social influence**					
		Subjective norm	N/A			
		Descriptive norm	N/A			
	Perceived security		0.15 (0.07)		.15 (.04)	
	**Prior usage experience**					
		Behavior regarding internet use	N/A			
		Frequency of smartphone use	N/A			
		Use of app for their diabetes	N/A			
	**Perceived health**					
		Attitude regarding diabetes self-management	N/A			
		Self-efficacy regarding diabetes self-management	N/A			
		Self-care regarding diabetes self-management	N/A			
	**Predictors regarding the use of the Iris app**					
		Perceived ease of use	0.40 (0.11)		.32 (<.001)	
	**Perceived social influence**					
		Subjective norm	0.31 (0.10)		.26 (.004)	
	Perceived control		0.16 (0.07)		.19 (.03)	
	Willingness to share data		0.22 (0.08)		.20 (.01)	

^a^*R*^2^=0.582. Step 3 multivariate analysis was performed with only the significant predictors of step 2.

^b^Not applicable (ie, variable was not significant in the previous step and thus not included in the analyses).

## Discussion

### Principal Findings

In this study we examined which factors could explain acceptance of a diabetes self-management eCoach (the Iris app). Acceptance of the app was mainly predicted by perceived usefulness, positive subjective norms, and willingness to share data. In addition, perceived usefulness was predicted by perceived security, positive subjective norms, willingness to share data, perceived ease of use, and perceived control. These predictors explained a high variance in acceptance and perceived usefulness of the Iris app (75.8% and 58.2%, respectively). Moreover, the TAM predictors regarding the use of the Iris app were more strongly associated with acceptance of this app than the predictors regarding the general usage of apps, smartphone, and internet. This can be explained by the compatibility principle, which indicates that the outcome variable will be better predicted if the specificity of the predictor matches the specificity of the outcome [46]. Furthermore, our study included elderly people (mean age of our sample was 63 years) and those with low educational level (40/92, 44%). Previous studies implied that elderly people and people with low SES may be more reluctant to use mHealth as they encounter more barriers while using innovative mHealth technologies [4,47]. Our findings showed that this was not the case regarding the Iris app, as there was no association between the sociodemographic factors (gender, age, education level, ethnicity, household) and the acceptance or perceived usefulness of the app.

Regarding the 2 core TAM predictors, perceived usefulness was strong and positively associated with acceptance of the Iris app, whereas perceived ease of use was not associated with acceptance of the app. However, perceived ease of use was moderately related to perceived usefulness of the app, which is in line with the TAM and a recent meta-analysis [48]. Moreover, perceived usefulness regarding the use of the (Iris) app showed to be a predictor but not perceived usefulness regarding the general use of apps, smartphone, and internet. Our findings imply that perceived usefulness is the most important predictor of acceptance of health technology, which is in line with previous studies (eg, [15,16,31,49]). Although our finding regarding perceived ease of use is in contrast to previous studies (eg, [31,49-51]), it was in line with the findings of Dou et al [16] and Zhang et al [15]. Their results did not show a significant association between perceived ease of use and patient’s intention to use smartphone health technology [16] or diabetes management apps [15], but rather implied an indirect association through perceived usefulness. Thus, if people feel the technology is easy to use, they would be more likely to develop positive attitudes toward the use of the health technology and perceive it as beneficial and helpful.

Besides the 2 core elements, the TAM has been extended with a range of other predictors. Our findings showed support for social influence as a predictor, but only for subjective norms specifically regarding the Iris app but not for subjective norms regarding the general use of mHealth technology (ie, apps, smartphone, and internet) nor descriptive norms. Thus, patients with T2D tend to do what is socially approved by other people regarding the use of the Iris app but not necessarily what is popular to do. A possible explanation as to why we did not find support for descriptive norms is that we did not measure this specifically regarding the app. Another possible explanation may be that, to assess descriptive norms, patients with T2D were only asked about the people they know. These people, however, are not necessarily the role models who patients with T2D acknowledge as important in their lives (eg, family, friends, health practitioner) or who they can identify with (eg, others who also have T2D). By contrast, to assess subjective norms, patients with T2D were explicitly asked about the people who are important in their lives (ie, family, friends, health practitioner).

Our findings showed that perceived behavioral control was not associated with acceptance of the Iris app by patients with T2D but was associated with perceived usefulness of the app. Individuals believing that they are capable of using the app are also more likely to perceive the app as beneficial and helpful. This finding is in line with extended models of the TAM [21,26] and with the findings of Dou et al [16]. They implied that perceived behavioral control was associated with perceived ease of use. In our study we did not examine this association, although we additionally calculated the correlation, which showed, in line with Dou et al [16], a significant, positive association (*r*=0.484, *P*<.001, n=92).

Our findings showed that willingness to share data predicted acceptance and perceived usefulness of the Iris app among patients with T2D. These findings imply that patients with T2D need to be convinced to share their app data. To do so, 2 issues might be important and need to be considered to increase the willingness of individuals to share their app data. First, some individuals (eg, older people, females, lower educated people, and people with a low propensity to trust others) are less willing to share information because they worry more about their privacy than others [37]. Our findings did not show a significant correlation between perceived security (including privacy) of health apps in general and sharing data of the Iris app (*r*=0.174, *P*=.10, n=92). Nevertheless, perceived security predicted perceived usefulness of the Iris app. However, more research is needed to examine which individuals are less likely to perceive security or share data on health technology use, and how these individuals can be convinced to share their data. Second, the data processing of the organization providing the mHealth tool can play an important role in convincing individuals to share information/data [37]. For example, the organization can consider the following aspects: (1) being transparent about the collection and storage of the data/information; (2) providing control to the individuals over whether or not their information/data are being collected, stored, and used; and (3) guaranteeing good security of the storage of the data/information [37].

### Limitations

Some limitations of this study also need to be addressed. First, this study used cross-sectional data. Therefore, we are not able to determine or provide interpretations of causality or possible predictors. Thus, although we talk about predictors, this needs to be interpreted with caution. Second, calculations in sample size software (Pass version 15) showed that our sample size achieves 80% power to detect a medium to large effect in the multiple regression analyses in step 2, with a significance level of .05. Furthermore, due to the sample size, we did not have sufficient power to test moderation effects of, for example, the sociodemographic factors (gender, age, education, ethnicity, household) or prior usage experience. Third, the majority of the measurements were self-constructed and based on the TAM, or empirical studies based on the TAM (eg, [16]), and the results of the 3 focus group interviews we conducted regarding the barriers and facilitators to use the Iris app among patients with diabetes. The reliability of the scales were good (Cronbach α ranged from .70 to .94). In addition, experts did check the face and content validity of the survey, but further validation of the survey is recommended. Fourth, although the explained variance was high in acceptance and perceived usefulness of the eCoach (75.8% and 58.2%, respectively), other important factors (eg, relationship to the doctor, resistance to change, enjoyment factor, technology anxiety, perceived value) might have contributed to an additional increase in the explained variance [16,52]. Moreover, for the final model (step 3), the scatterplots (see Multimedia Appendices 1 and 2) show that the model gives a pretty good prediction but that at the extremes there is a bit more dispersion in prediction. These other important factors might improve the model quality in future research. Finally, the participants that we recruited via online advertisement might also be the ones that often use mHealth technology, and thus, who perceive ease of use as less important. However, the descriptive statistics show this is probably not the case, as less than 40% (35/92, 38%) of the participants used an app for diabetes management.

### Implications for Practice and Future Research

Our findings regarding subjective norms imply that (general) health practitioners, family, and friends might play an important role in facilitating the acceptance and perceived usefulness of the Iris app. To successfully develop and introduce this app for diabetes management among patients with T2D (including elderly and lower educated people), it might be important to focus our efforts on blending the app with contact and communication with (general) health practitioners. For example, the health practitioner could play a role in discussing and encouraging the patient with T2D to share their app data, in explaining why the app could be beneficial/useful for managing their diabetes, and in communicating and guaranteeing the privacy when using the app. Moreover, family and friends increased the patients’ acceptance and perceived usefulness of the app through their approval of using the app, and therefore they perhaps could also play a role in discussing and encouraging the patient with T2D to use the app.

Although our findings indicated that the explained variance was high in acceptance and perceived usefulness of the Iris app (75.8% and 58.2%, respectively), future research might also examine other additional predictors that might be essential for the acceptance of a diabetes self-management eCoach (eg, relationship to the doctor, social relationship, resistance to change, personal innovativeness, enjoyment factor, technology anxiety, perceived value) [16,52,53]. Furthermore, to develop and introduce an app for diabetes self-management, future research should need to identify which features of the app are effective for which type of individuals [11].

### Conclusions

To stimulate the acceptance of a self-management eCoach for patients with T2D (including elderly people and lower educated people), we need to achieve that patients with T2D perceive the app as beneficial and helpful to use, and are willing to share their app data. In addition, we need to achieve that the people who are important in the life of patients with T2D (eg, family, friends, health practitioner) socially approve the use of this app. Furthermore, elderly and lower educated people were represented within our sample, but did not seem to score lower on acceptance of perceived usefulness of the app compared with younger people (<63 years old) or higher educated people (completing an intermediate or high level of education).

## Appendix

Multimedia Appendix 1 Scatterplot of acceptance of the Iris app against the predicted values.

Multimedia Appendix 2 Scatterplot of perceived usefulness of the Iris app against the predicted values.

## Abbreviations

DMSES: Diabetes Management Self-Efficacy Scale
DSMQ: Diabetes Self-Management Questionnaire
eCoach: electronic coach
IRB: institutional review board
mHealth: mobile health
N/A: not applicable
SES: socioeconomic status
STAM: Senior Technology Acceptance Model
T2D: type 2 diabetes
TAM: Technology Acceptance Model
TNO: Netherlands Organization for Applied Scientific Research
UTAUT: Unified Theory of Acceptance and Use of Technology
