# Supplementary figures and images for: Predictors of the Acceptance of an Electronic Coach Targeting Self-management of Patients With Type 2 Diabetes: Web-Based Survey
Source: JMIR Form Res. 2022 Aug 16;6(8):e34737. doi: 10.2196/34737 (PMC9428778; doi:10.2196/34737)

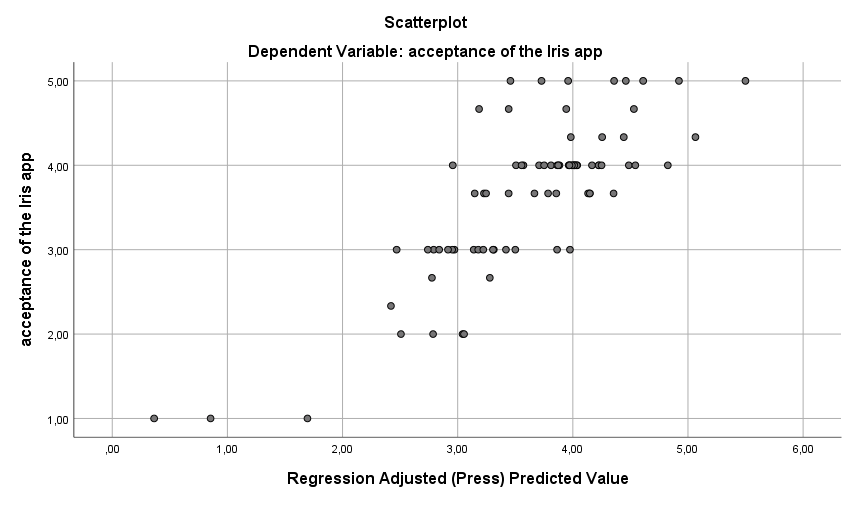

Supplement: Multimedia Appendix 1 [file formative_v6i8e34737_app1.png]

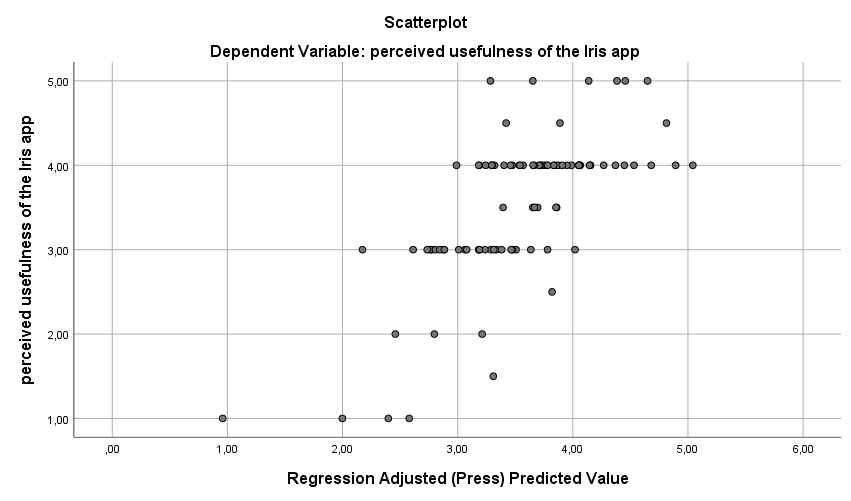

Supplement: Multimedia Appendix 2 [file formative_v6i8e34737_app2.png]
